# LncRNA NR2F2‐AS1 inhibits the progression of oral squamous cell carcinoma by mediating the miR‐32‐5p/SEMA3A axis

**DOI:** 10.1002/kjm2.12888

**Published:** 2024-08-23

**Authors:** Shi‐Yu Qin, Bo Li, Ji‐Mu Liu, Qiu‐Li Lv, Xiang‐Lin Zeng

**Affiliations:** ^1^ Department of Oral and Maxillofacial Surgery Affiliated Stomatology Hospital of Guilin Medical University Guilin Guangxi Zhuang Autonomous Region P.R. China

**Keywords:** angiogenesis, miR‐32‐5p, NR2F2‐AS1, OSCC, SEMA3A

## Abstract

Previous studies have supported a tumor‐suppressive role of semaphorin 3A (SEMA3A) in several tumors including oral squamous cell carcinoma (OSCC). However, in‐depth characterization of the role of SEMA3A in OSCC and the underlying molecular mechanisms is lacking. Gene and protein expressions were detected using quantitative real‐time PCR, western blot assay, and immunohistochemistry. OSCC cell metastasis was evaluated using Transwell and angiogenesis of human umbilical vein endothelial cells (HUVECs) was determined using tube formation assay. The interactions among molecules were predicted using bioinformatics analysis and validated using luciferase activity experiment and RNA immunoprecipitation assay. Pulmonary metastasis was evaluated using hematoxylin and eosin staining after constructing a lung metastasis tumor model in mice. SEMA3A expression was decreased in OSCC cells and its overexpression led to suppression of epithelial‐mesenchymal transition (EMT), migration, and invasion of OSCC cells and angiogenesis of HUVECs. miR‐32‐5p was identified as an upstream molecule of SEMA3A and long non‐coding RNA NR2F2 antisense RNA 1 (NR2F2‐AS1) was validated as an upstream gene of miR‐32‐5p. Further experiments revealed that the inhibitory effects of NR2F2‐AS1 overexpression on EMT, migration, invasion of OSCC cells, and angiogenesis of HUVECs as well as tumor growth and metastasis in mice were mediated via the miR‐32‐5p/SEMA3A axis. To conclude, NR2F2‐AS1 may attenuate OSCC cell metastasis and angiogenesis of HUVECs and suppress tumor growth and metastasis in mice via the miR‐32‐5p/SEMA3A axis.

## 1 INTRODUCTION

Oral cancer is associated with high morbidity and mortality. In 2020, more than 370,000 new cases of lip and oral cavity cancer and over 70,000 deaths were reported worldwide.^1^ Oral squamous cell carcinoma (OSCC) accounts for >90% of all cases of oral cancer.^2^ The reported 5‐year overall survival (OS) rate of patients with OSCC is 50%–60%. Although the OS rate is relatively high (approximately 80%) in the early stage, it is as low as 20%–40% in the late stage.^3^, ^4^ Identification of novel tumor markers for OSCC and their underlying molecular regulatory mechanisms is a contemporary research hotspot.^5^ This study aimed to identify molecules that play a key role in OSCC progression and explore their molecular regulatory mechanisms.

The semaphorins (SEMA) family comprises largely of membrane proteins and secreted proteins with a common “SEMA” structure.^6^ Studies have demonstrated the involvement of SEMA family members including eight subtypes in various physiological and pathological processes such as neurogenesis, organ formation, angiogenesis, immune response, and tumor growth.^6^ Semaphorin 3A (SEMA3A) is a key signaling protein in the development of nerve axons, and growing evidence now points to its significant role in the field of oncology.^7^ For instance, Luo et al. reported that SEMA3A, negatively regulated by miR‐362, delayed metastasis of lung cancer.^8^ Several studies have suggested the inhibitory effect of SEMA3A on malignant characteristics and progression of OSCC.^9^ However, the underlying molecular mechanisms of the effects of SEMA3A in OSCC are unclear.

MicroRNAs (miRNAs) are a class of non‐coding single‐stranded RNA molecules, with a length of about 22 nucleotides. miRNAs play a role in the development of various diseases including OSCC.^7^ miR‐32‐5p has been implicated in multiple tumors including prostate cancer, pancreatic cancer, and OSCC.^10^ For instance, miR‐32‐5p was shown to promote the epithelial‐to‐mesenchymal transition of OSCC.^10^ Additionally, the 3′‐UTR region of mRNA is complementary and paired with miRNA to form an RNA‐induced silencing complex (RISC), which results in the degradation of mRNA to gene expression.^11^ For example, miR‐26a‐5p was shown to interact with SLC7A11 mRNA to inhibit its expression, thereby enhancing ferroptosis in OSCC.^11^ Long non‐coding RNAs (lncRNAs) are non‐coding RNAs with a length greater than 200 nucleotides, which also play a role in several diseases including OSCC.^12^ LncRNA NR2F2 antisense RNA 1 (NR2F2‐AS1) was reported to have a promotive or suppressive effect in various cancers, such as non‐small cell lung cancer (NSCLC) and OSCC.^12^, ^13^ Of note, only one study has reported the downregulation of NR2F2‐AS1 expression in OSCC and its inhibitory effect on cell proliferation.^12^ Besides, the lncRNA‐miRNA‐mRNA signal axis has been extensively studied in oncology and OSCC progression.^14^, ^15^ For instance, the lncRNA DANCR/miR‐216a‐5p/KLF12 axis was shown to be involved in cell growth and metastasis of OSCC.^16^ Currently, the effect of NR2F2‐AS1 on the malignant behavior of OSCC cells and the underlying molecular regulatory mechanisms are not clear.

Based on the above‐mentioned evidence and some preliminary experiments, we hypothesized that NR2F2‐AS1 could suppress the epithelial‐mesenchymal transition (EMT), migration, invasion of OSCC cells, and angiogenesis of human umbilical vein endothelial cells (HUVECs) and attenuate tumor growth and metastasis of OSCC in vivo through the miR‐32‐5p/SEMA3A axis.

## 2 METHODS

### 2.1 Cell culture

Human OSCC cell lines (HSC3, SCC9, SCC15, and SCC25), human immortalized epidermal cells (HaCaT), and HUVECs were used in this study. SCC9, SCC15, SCC25, and HUVEC were acquired from ATCC (USA), HSC3 cells were purchased from JCRB Cell Bank (Japan), and HaCaT was acquired from Cosmo Bio Co. Ltd. (Japan). The above‐mentioned cells, except for HUVECs, were incubated in DMEM medium (Thermo Fisher Scientific, USA) supplemented with 10% FBS (Thermo Fisher Scientific). HUVECs were cultured in endothelial growth medium, which was supplemented with endothelial basal medium, human recombinant epidermal growth factor, EndoCGS‐Heparin (PeloBiotech, Germany), and 8% fetal calf serum (Biochrom, Germany). The medium was supplemented with 1% antibiotics (Beyotime, China), and the cells were placed in an incubator under 5% CO_2_ and at 37°C.

### 2.2 Cell transfection

To overexpress SEMA3A and knock down miR‐32‐5p, pcDNA3.1‐SEMA3A and miR‐32‐5p inhibitors as well as corresponding controls were acquired from GenePharma (China). Additionally, to overexpress NR2F2‐AS1 expression and to silence SEMA3A, OSCC cells were transfected with lentivirus carrying NR2F2‐AS1 or sh‐SEMA3A. The target sequences of sh‐SEMA3A and sh‐NC were as follows: sh‐SEMA3A: 5′‐CGGTGTGATATTTACGGGAAA‐3′; sh‐NC: 5′‐GTTCTCCGAACGTGTCACGT‐3′. Following the instructions, OSCC cells were transfected with plasmids for 48 h using Lipofectamine™ 3000 (Invitrogen, USA). For lentivirus infection, OSCC cells were infected with lentivirus carrying NR2F2‐AS1 or sh‐SEMA3A (purchased from GenePharma) with the aid of polybrene. Puromycin was applied for screening infected OSCC cells after 72 h.

### 2.3 Quantitative real‐time PCR (RT‐qPCR)

The expression of targeted genes was determined using RT‐qPCR. In brief, total RNA was extracted from cells and mice tumors using TRIzol reagent (Beyotime, China) and miRNA was extracted using PureLink™ miRNA isolation kit (Thermo Fisher Scientific). The extracted RNA were reverse‐transcribed into cDNA using Script Reverse Transcription Reagent kit (TaKaRa, China). SYBR Premix Ex Taq II kit (TaKaRa) was used for the RT‐qPCR process. The primer sequences were obtained from Sangon (China) (Table 1). After amplification by primer, the relative expression of targeted genes was calculated using the 2^−ΔΔCt^ formula. GAPDH and U6 served as reference genes.

**TABLE 1:** Primer sequences of genes.

Gene name	Forward	Reverse
SEMA3A	5′‐TGGTTCTGCATGTTCTCGCT‐3′	5′‐AATAGACCAGCGCTCTCTGC‐3′
miR‐32‐5p	5′‐GCCGAGTATTGCACATTACTAA‐3′	5′‐GTCGTATCCAGTGCAGGGTCCGAGGTATTCGCACTGGATACGACTGCAAC‐3′
NR2F2‐AS1	5′‐GTGAGTCGGAGCTCCTGCAG‐3′	5′‐GGAGATGCCGGGCATTGTGG‐3′
U6	5′‐CTCGCTTCGGCAGCACA‐3′	5′‐AACGCTTCACGAATTTGCGT‐3′
GAPDH	5′‐CCAGGTGGTCTCCTCTGA‐3′	5′‐GCTGTAGCCAAATCGTTGT‐3′

### 2.4 Western blot

Total proteins from OSCC cells with various treatments were extracted and the concentration of proteins was determined using a BCA protein concentration determination kit (Beyotime). Proteins were isolated using electrophoresis and transferred onto PVDF membranes. After blocking by skimmed milk (5%), membranes were incubated overnight at 4°C with the following primary antibodies: anti‐E‐cadherin (14‐3249‐82, 1:200), anti‐N‐cadherin (33‐3900, 1:1000), anti‐Vimentin (MA5‐11883, 1:1000), anti‐SEMA3A (PA5‐118891, 1:2000), and anti‐GAPDH (MA1‐16757, 1:1000), which were purchased from Thermo Fisher Scientific. Then, membranes were immersed with HRP‐conjugated secondary antibody (Beyotime) for 1 h. Finally, protein bands were visualized using the ECL kit (Beyotime), and the gray values of protein bands were evaluated by ImageJ.

### 2.5 Transwell assay

Transwell assay was performed to evaluate cell migration and invasion using a 24‐well Transwell insert system (Corning, USA). OSCC cells with the indicated treatments were inoculated in the top chamber, which was added with serum‐free DMEM. Additionally, DMEM containing 10% FBS was added to the lower chamber. After 24 h, cells migrated to the below side of the chamber were fixed with 95% alcohol, stained with 1% crystal violet (Sigma‐Aldrich, USA), and examined under an optical microscope (Olympus, Japan). The top chamber of the Transwell insert system was covered with Matrigel (Becton Dickinson Biosciences, USA) for detecting cell invasion. Other steps were consistent with the steps for detecting cell migration.

### 2.6 Tube formation assay

To evaluate the effect of conditioned medium (CM) of OSCC cells on the angiogenesis of HUVECs, matrix glue (50 μL/well) was added into a 96‐well plate and placed in an incubator at 37°C for 45 min. Subsequently, HUVECs were suspended in CM of OSCC cells, and 50 μL suspension including approximately 3 × 10^4^ HUVECs was added into each well of the 96‐well plate. After 6 h, the angiogenesis of HUVEC was observed under an optical microscope.

### 2.7 Bioinformatics analysis

To predict upstream miRNAs that regulate SEMA3A expression, Starbase (https://rnasysu.com/encori/), miRDB (https://mirdb.org/), and miRWalk (http://mirwalk.umm.uni-heidelberg.de/) websites were used.

LncBook (https://ngdc.cncb.ac.cn/lncbook/) and lncRNASNP 2 (https://guolab.wchscu.cn/lncRNASNP/#!/) databases were employed to predict upstream lncRNAs that regulate miR‐32‐5p expression.

### 2.8 Luciferase activity experiment

The interactions between SEMA3A 3′‐UTR and miRNAs including miR‐32‐5p, miR‐92a‐3p, and miR‐320c, and the interaction between miR‐32‐5p and NR2F2‐AS1 were validated using dual luciferase reporter assay. SEMA3A 3′‐UTR wild type (SEMA3A‐wt), mutant (SEMA3A‐mut), NR2F2‐AS1‐wt, NR2F2‐AS1‐mut, miR‐32‐5p mimic, miR‐92a‐3p mimic, miR‐320c mimic, and miR‐NC were obtained from GenePharma. For validating interactions between SEMA3A 3′‐UTR and miRNAs (miR‐32‐5p, miR‐92a‐3p, and miR‐320c), miR‐32‐5p mimic, miR‐92a‐3p mimic, or miR‐320c mimic/miRNAs‐NC in combination with SEMA3A‐wt/SEMA3A‐mut were transfected into OSCC cells using Lipofectamine 3000 (Invitrogen, USA). To verify the interaction between miR‐32‐5p and NR2F2‐AS1, miR‐32‐5p mimic/miR‐NC together with NR2F2‐AS1‐wt/NR2F2‐AS1‐mut were transfected into OSCC cells using Lipofectamine 3000. Finally, luciferase activity was evaluated using the dual luciferase reporter system kit (Promega, USA).

### 2.9 RNA immunoprecipitation (RIP) assay

OSCC cells were lysed with RIP lysis buffer. The supernatants were obtained after centrifugation. The magnetic beads conjugated with antibodies anti‐AGO2 (ab186733, Abcam) or IgG (ab172730, Abcam, UK) were added to the supernatants. IgG acted as the control. The beads‐bound complexes of RNA were eluted with elution buffer. Then, immune‐precipitated RNAs were determined using RT‐qPCR.

### 2.10 RNA pulldown

RNA pulldown was conducted to screen out the lncRNAs interacting with miR‐32‐5p. SCC9 cells were transfected with biotin‐miR‐32‐5p for 48 h. Dynabeads M‐280 Streptavidin‐coated magnetic beads (Thermo Fisher Scientific) were activated according to the manufacturer's protocol. Then, the beads were washed with lysis buffer. SCC9 cells were incubated with lysis buffer after washing. The lysates were incubated overnight with streptavidin‐coated magnetic beads at 4°C. The RNAs obtained by the pulldown of biotin‐miR‐32‐5p were detected by agarose gel electrophoresis.

### 2.11 Nucleocytoplasmic separation experiment

Nuclear and cytoplasmic RNAs of SCC9 cells were separated using PARIS™ kit (Invitrogen, USA). Cytoplasmic lysis buffer was added to suspended cell pellets and the collected supernatant served as the cytosol fraction. In addition, cells were lysed with nuclear lysis buffer and the collected supernatant served as the nuclear fraction. After purification and DNase I (Solarbio, China) treatment, nuclear and cytoplasmic RNAs were reverse‐transcribed into cDNAs. The expression of NR2F2‐AS1 in the cytoplasm and nucleus was detected by RT‐qPCR.

### 2.12 Detection of tumor formation and pulmonary metastasis in nude mice

Nude mice aged 3–5 weeks were purchased from Hunan Slac Jingda Laboratory Animal Co. Ltd. (China) and were randomly assigned to control, lenti‐vector, lenti‐NR2F2‐AS1, and lenti‐NR2F2‐AS1 + sh‐SEMA3A groups (6 mice per group). In the lenti‐NR2F2‐AS1 group, SCC9 cells were infected with lentivirus carrying NR2F2‐AS1, and in the NR2F2‐AS1 + sh‐SEMA3A group, SCC9 cells were infected with lentivirus carrying NR2F2‐AS1 and sh‐SEMA3A. The transfected SCC9 cells (2 × 10^6^ cells) were subcutaneously inoculated into the axilla of the left forelimb of nude mice. After cell inoculation, tumor volume was calculated every 5 days until 30 days using the formula: tumor volume = length × width^2^/2. After 30 days, mice were euthanized and tumor tissues were collected and subjected to immunohistochemistry (IHC) and RT‐qPCR.

For pulmonary metastasis, nude mice were administered an intravenous injection of 5 × 10^5^ SCC9 cells with the above‐mentioned transfection through the tail vein. After 30 days, nude mice were euthanized, and the lung tissues were carefully removed. The lung tissues were photographed and the pulmonary nodules were observed. In addition, hematoxylin and eosin (HE) staining was used to examine the lung morphology. Animal experiments were approved by the ethics committee of Affiliated Stomatological Hospital of Guilin Medical College.

### 2.13 IHC experiment

IHC was conducted to examine SEMA3A and CD31 expression in tumor tissues of mice. Tumor tissues were fixed with 4% PFA, paraffin‐embedded, and cut into 5‐μm thick sections. The sections were blocked with 1% BSA after antigen repair. Subsequently, the sections were incubated overnight with anti‐SEMA3A (Thermo Fisher Scientific) and anti‐CD31 (Thermo Fisher Scientific) at 4°C. Next, the sections were incubated with HRP‐labeled antibody (Abcam) and diaminobenzidine (DAB) in turn. Finally, the images were examined using an optical microscope.

### 2.14 HE staining

HE‐stained sections of mice lung tissue were examined to observe histomorphology. In brief, lung tissues were fixed using 4% paraformaldehyde. After paraffin embedding, 5 μm sections were obtained. The sections were dehydrated using ethanol, stained with HE, and examined using an optical microscope.

### 2.15 Statistical analysis

All data were analyzed using GraphPad Prism 9. Data were presented as mean ± standard deviation (SD). Between‐group differences were assessed for statistical significance using Student's *t*‐test. One‐way analysis of variance (ANOVA) followed by Tukey's post hoc test was used for multi‐group comparisons. *p* < 0.05 was considered indicative of statistical significance.

## 3 RESULTS

### 3.1 SEMA3A upregulation inhibited EMT, migration, and invasion of OSCC cells and angiogenesis of HUVECs

**FIGURE 1:**
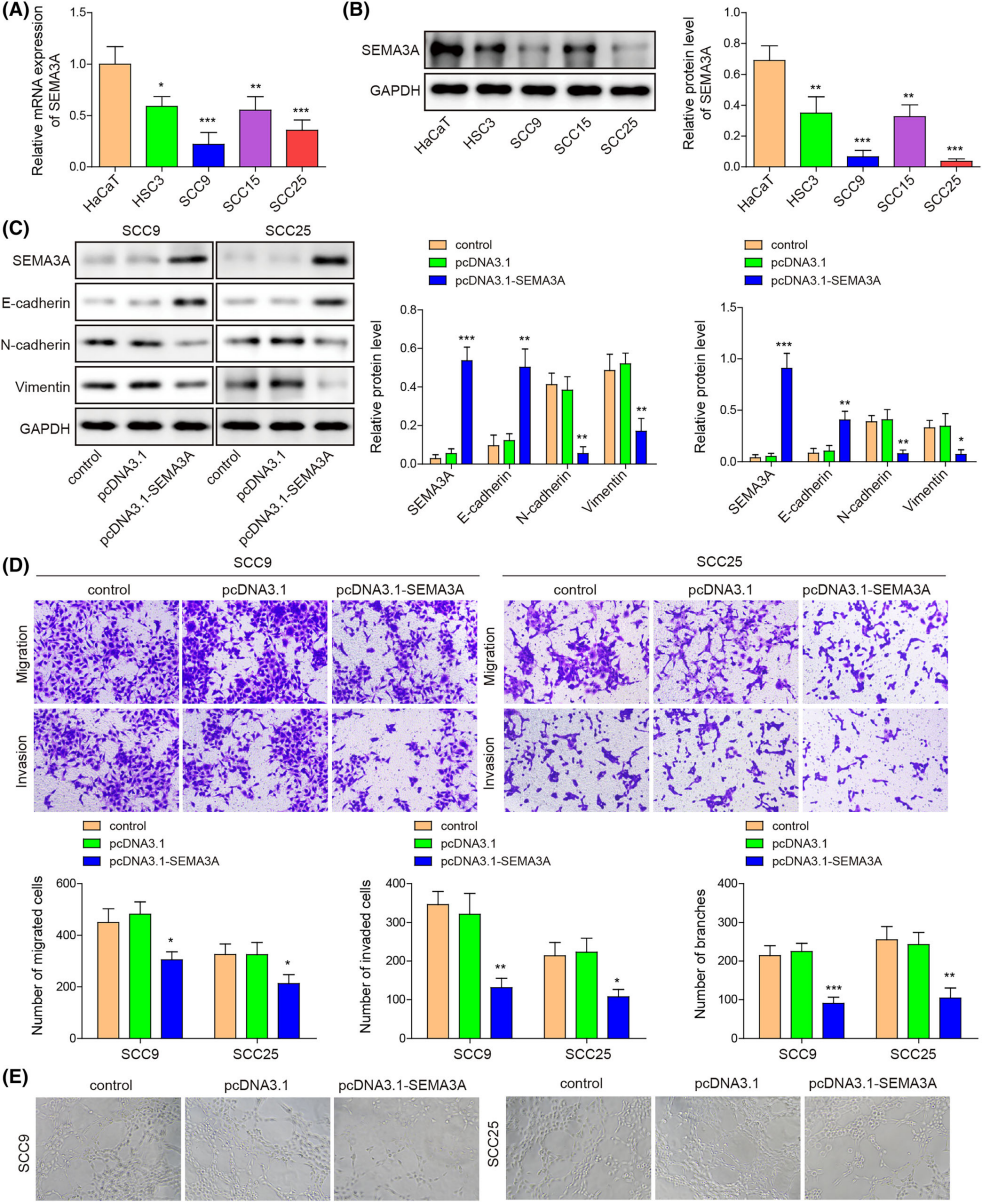
SEMA3A upregulation impaired EMT, migration, and invasion of OSCC cells and angiogenesis of HUVECs. (A and B) SEMA3A expression in HaCaT, HSC3, SCC9, SCC15, and SCC25 cells was evaluated using RT‐qPCR and Western blot assay. SCC9 and SCC25 cells were subjected to pcDNA3.1‐SEMA3A transfection. (C) SEMA3A, E‐cadherin, N‐cadherin, and Vimentin expression were detected by Western blot assay. (D) Cell migration and invasion were measured using Transwell assay. HUVECs were treated with medium supernatants, which were obtained from OSCC cells with the indicated transfection. (E) Angiogenesis of HUVECs was detected by tube formation assay. *p < 0.05, **p < 0.01, ***p < 0.001. EMT, epithelial‐mesenchymal transition; HUVECs, human umbilical vein endothelial cells; OSCC, oral squamous cell carcinoma; RT‐qPCR, quantitative real‐time PCR; SEMA3A, semaphorin 3A.

We compared SEMA3A expression in human immortalized epidermal cells (HaCaT) and four OSCC cells (HSC3, SCC9, SCC15, and SCC25). We observed significantly decreased expression of SEMA3A in OSCC cells, especially in SCC9 and SCC25 cells (Figure 1A,B). To assess the influence of SEMA3A on malignant characteristics of OSCC cells, SEMA3A expression was upregulated in SCC9 and SCC25 cells using pcDNA3.1‐SEMA3A transfection and was downregulated using sh‐SEMA3A transfection, which was confirmed by Western blot assay (Figure 1C and Figure S1A). SEMA3A overexpression in SCC9 and SCC25 cells delayed the EMT process, as indicated by increased E‐cadherin expression and decreased N‐cadherin and Vimentin expression (Figure 1C). SEMA3A knockdown had the opposite effect (Figure S1A). Furthermore, OSCC cell migration and invasion were attenuated by SEMA3A overexpression while enhanced by SEMA3A knockdown (Figure 1D and Figure S1B). Subsequently, medium supernatants from OSCC cells with the indicated transfection were added into HUVECs, and, as expected, SEMA3A upregulation also suppressed angiogenesis of HUVECs whereas SEMA3A knockdown promoted angiogenesis (Figure 1E and Figure S1C). Collectively, these findings indicated that SEMA3A upregulation inhibited malignant behaviors of OSCC cells.

### 3.2 miR‐32‐5p decreased SEMA3A expression via interacting with SEMA3A

**FIGURE 2:**
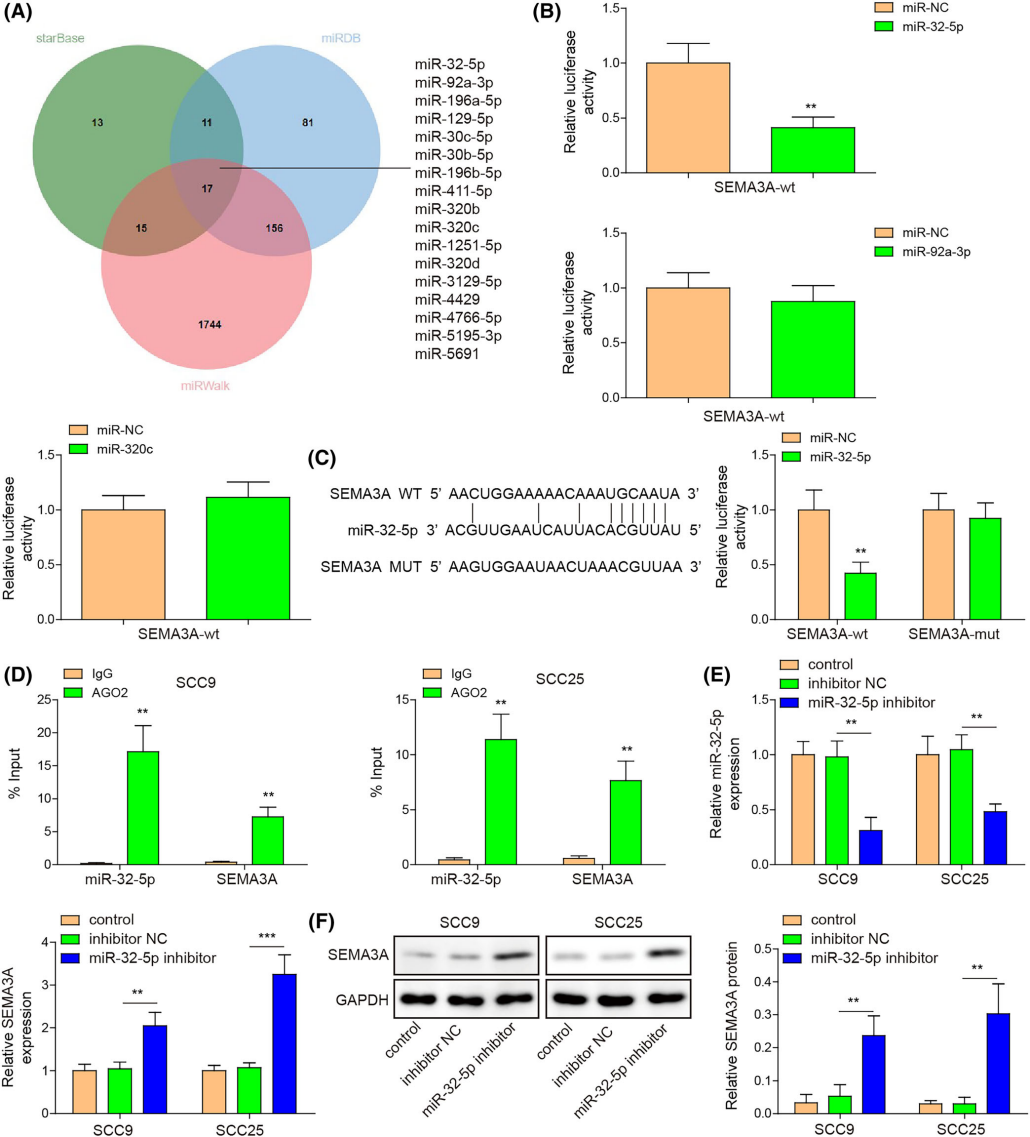
miR‐32‐5p decreased SEMA3A expression by interacting with SEMA3A. (A) Overlapping miRNAs targeting SEMA3A were obtained from Starbase, miRDB, and miRWalk databases. (B) miR‐32‐5p, miR‐92a‐3p, and miR‐320c targeting SEMA3A were verified by dual luciferase assay. (C and D) The binding sequences between miR‐32‐5p and SEMA3A were presented and the interaction between miR‐32‐5p and SEMA3A was validated by dual luciferase assay and RIP assay. (E) miR‐32‐5p and SEMA3A expression were measured in OSCC cells transfected with inhibitor NC or miR‐32‐5p inhibitor using RT‐qPCR. (F) SEMA3A protein expression was measured in OSCC cells transfected with inhibitor NC or miR‐32‐5p inhibitor using Western blot assay. **p < 0.01, ***p < 0.001. OSCC, oral squamous cell carcinoma; RIP, RNA immunoprecipitation; RT‐qPCR, quantitative real‐time PCR; SEMA3A, semaphorin 3A.

miRNAs are known to regulate target gene expression by complementary pairing with the 3′‐UTR region of mRNAs.^11^ Therefore, we employed three bioinformatics databases including Starbase, miRDB, and miRWalk to predict potential miRNAs binding to SEMA3A. Starbase, miRDB, and miRWalk predicted 56, 265, and 1932 potential miRNAs, respectively. Of these, 17 overlapping miRNAs (miR‐32‐5p, miR‐92a‐3p, miR‐196a‐5p, miR‐129‐5p, miR‐30c‐5p, miR‐30b‐5p, miR‐196b‐5p, miR‐411‐5p, miR‐320b, miR‐320c, miR‐1251‐5p, miR‐320d, miR‐3129‐5p, miR‐4429, miR‐4766‐5p, miR‐5195‐3p, and miR‐5691) were identified through jvenn analyses (Figure 2A). In addition, we provided the targeting relationship scores of 17 miRNAs, which were predicted by the miRDB database (Table S1). miR‐32‐5p, miR‐92a‐3p, and miR‐320c have been reported to be over‐expressed in OSCC.^10^, ^17^ However, other miRNAs were either reported to exhibit decreased expression in OSCC or were not reported in published studies. The specific query results of 17 miRNAs are provided in Table S2. Subsequently, these three miRNAs were employed for dual luciferase assay, which was used to validate the interaction between miRNAs and SEMA3A. As presented in Figure 2B, there was a targeting relationship between miR‐32‐5p and SEMA3A, but not between miR‐92a‐3p/miR‐320c and SEMA3A. Figure 2C exhibits the binding sequences between miR‐32‐5p and SEMA3A; miR‐32‐5p mimics resulted in decreased luciferase activity in SEMA3A‐wt, but failed in SEMA3A‐mut. Additionally, the RIP assay displayed that AGO2 successfully enriched miR‐32‐5p and SEMA3A (Figure 2D). Moreover, miR‐32‐5p inhibitor caused a reduction of miR‐32‐5p expression and enhancement of SEMA3A expression in OSCC cells (Figure 2E,F). Collectively, these findings indicated that miR‐32‐5p targeted SEMA3A and negatively regulated SEMA3A expression.

### 3.3 NR2F2‐AS1 interacted with miR‐32‐5p

**FIGURE 3:**
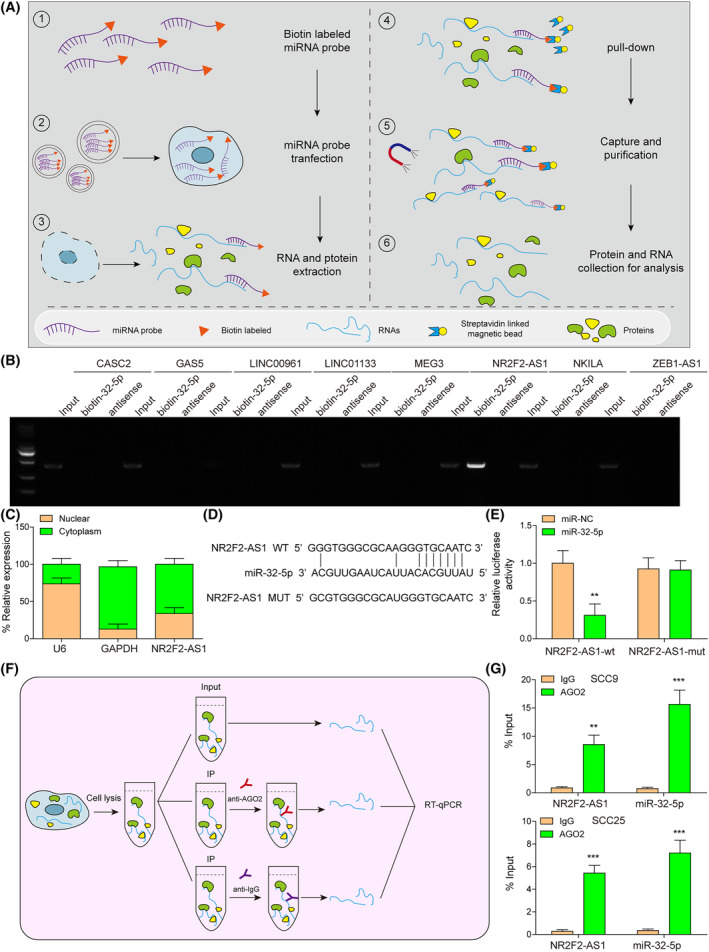
NR2F2‐AS1 interacted with miR‐32‐5p. (A) The experimental procedure of RNA pulldown. (B) The interaction between CASC2/GAS5/LINC00961/LINC01133/MEG3/NR2F2‐AS1/NKILA/ZEB1‐AS1 and miR‐32‐5p was validated by RNA pulldown. (C) The location of NR2F2‐AS1 was determined using nucleocytoplasmic separation experiment. (D) The binding sequences between NR2F2‐AS1 and miR‐32‐5p were predicted by the lncRNASNP database. (E) The interaction between NR2F2‐AS1 and miR‐32‐5p was validated by dual luciferase assay. (F) The experimental procedure of RIP. (G) The interaction between NR2F2‐AS1 and miR‐32‐5p was validated by RIP assay. **p < 0.01, ***p < 0.001. RIP, RNA immunoprecipitation.

Several studies have shown that lncRNAs modulate the expression of mRNAs via sponging miRNAs.^18^ Based on published studies, we identified 21 lncRNAs with downregulated expression in OSCC, which are presented in Table S3. According to prediction using LncBook and lncRNASNP 2 databases, eight of these lncRNAs (CASC2, GAS5, LINC00961, LINC01133, MEG3, NR2F2‐AS1, NKILA, and ZEB1‐AS1) were found to have potential binding relationships with miR‐32‐5p. Therefore, RNA pulldown was conducted to validate interactions of the above lncRNAs with miR‐32‐5p. Figure 3A exhibits the experimental procedure of RNA pulldown. In the pulldown products of miR‐32‐5p, NR2F2‐AS1 was detected, but other lncRNAs were not detected, indicating the interaction of NR2F2‐AS1 with miR‐32‐5p in SCC9 cells (Figure 3B). Besides, the results of the nucleocytoplasmic separation experiment showed that NR2F2‐AS1 was mainly localized in the cytoplasm (Figure 3C). Furthermore, the interaction between NR2F2‐AS1 and miR‐32‐5p was validated using dual luciferase assay and RIP assay. Figure 3D exhibits the binding sequences between NR2F2‐AS1 and miR‐32‐5p, which were predicted by the lncRNASNP 2 database. Additionally, miR‐32‐5p mimics suppressed luciferase activity in NR2F2‐AS1‐wt rather than NR2F2‐AS1‐mut (Figure 3E). Furthermore, a RIP assay was conducted to validate the interaction between NR2F2‐AS1 and miR‐32‐5p. The experimental procedure of RIP is presented in Figure 3F. The results showed that AGO2 obtained enrichment of NR2F2‐AS1 and miR‐32‐5p (Figure 3G). These findings indicate that NR2F2‐AS1 interacted with miR‐32‐5p.

### 3.4 NR2F2‐AS1 suppressed the EMT, migration, invasion of OSCC cells, and the angiogenesis of HUVEC via the miR‐32‐5p/SEMA3A axis

**FIGURE 4:**
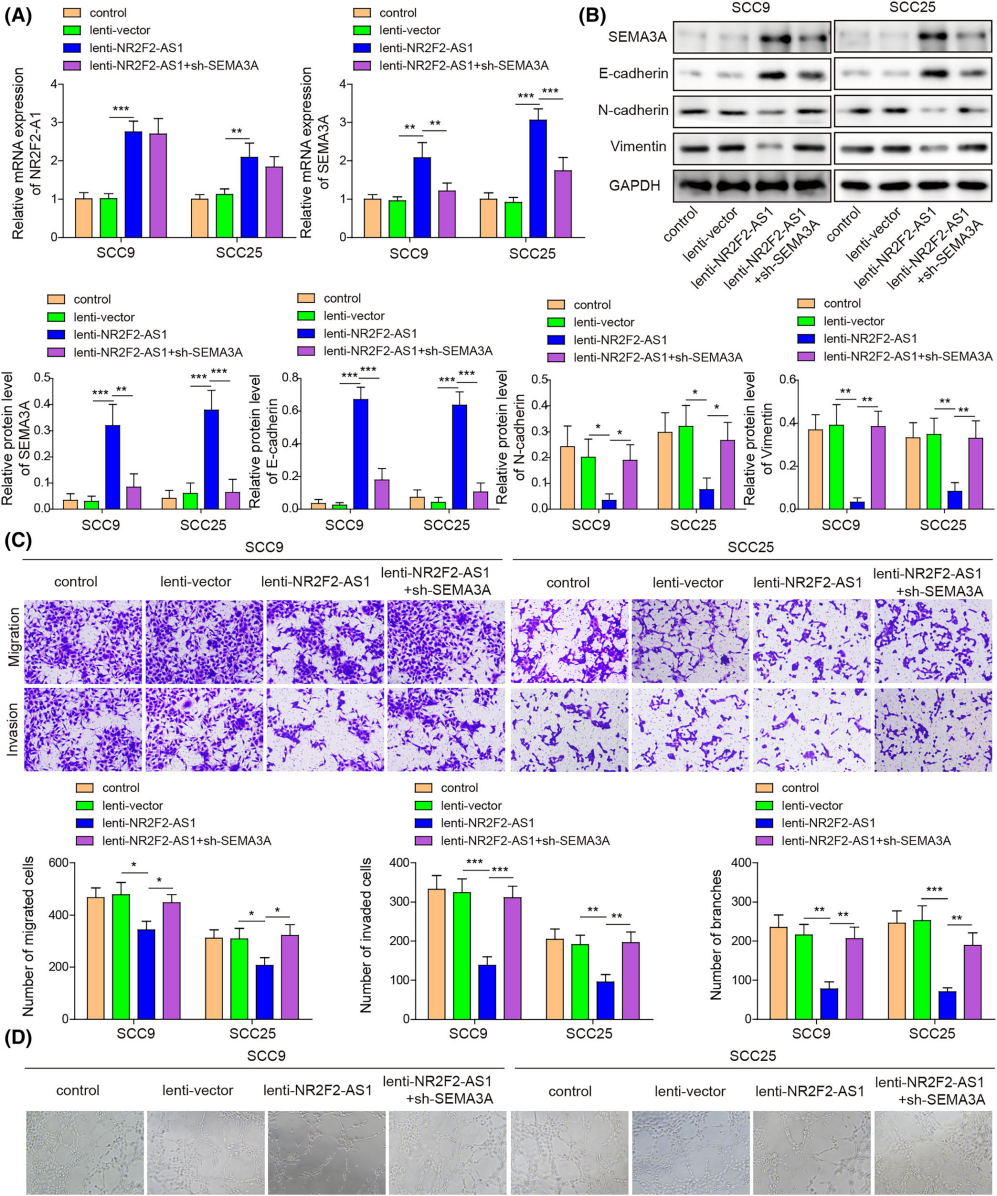
NR2F2‐AS1 suppressed EMT, migration, and invasion of OSCC cells and the angiogenesis of HUVECs via the miR‐32‐5p/SEMA3A axis. SCC9 and SCC25 cells were infected with lentivirus delivering NR2F2‐AS1 or together with sh‐SEMA3A. (A) NR2F2‐AS1 and SEMA3A expression were evaluated by RT‐qPCR. (B) SEMA3A, E‐cadherin, N‐cadherin, and Vimentin expression were detected by Western blot assay. (C) Cell migration and invasion were measured using Transwell assay. HUVECs were treated with medium supernatants, which were obtained from OSCC cells with the indicated transfection. (D) Angiogenesis of HUVECs was detected by tube formation assay. *p < 0.05, **p < 0.01, ***p < 0.001. EMT, epithelial‐mesenchymal transition; HUVECs, human umbilical vein endothelial cells; OSCC, oral squamous cell carcinoma; RT‐qPCR, quantitative real‐time PCR; SEMA3A, semaphorin 3A.

To investigate whether NR2F2‐AS1/miR‐32‐5p/SEMA3A axis influences EMT, migration, invasion, and angiogenesis of OSCC, SCC9 and SCC25 cells were infected with lentivirus carrying NR2F2‐AS1 or together with sh‐SEMA3A. As shown in Figure 4A,B, NR2F2‐AS1 overexpression increased NR2F2‐AS1 and SEMA3A expression, while SEMA3A silencing abolished NR2F2‐AS1 overexpression‐mediated upregulation of SEMA3A expression. NR2F2‐AS1 upregulation suppressed EMT process, as evidenced by elevated E‐cadherin expression and reduced N‐cadherin and Vimentin expression in SCC9 and SCC25 cells. SEMA3A inhibition compromised these alterations caused by NR2F2‐AS1 upregulation (Figure 4B). Besides, migration and invasion of OSCC cells were inhibited by NR2F2‐AS1 overexpression, which was reversed by SEMA3A knockdown (Figure 4C). NR2F2‐AS1 overexpression also decreased angiogenesis of HUVECs, which was compromised by SEMA3A silencing (Figure 4D). Collectively, these findings demonstrate that NR2F2‐AS1 overexpression attenuated malignant features of OSCC cells by elevating SEMA3A expression.

### 3.5 NR2F2‐AS1 impeded the growth and metastasis of OSCC in vivo via the miR‐32‐5p/SEMA3A axis

**FIGURE 5:**
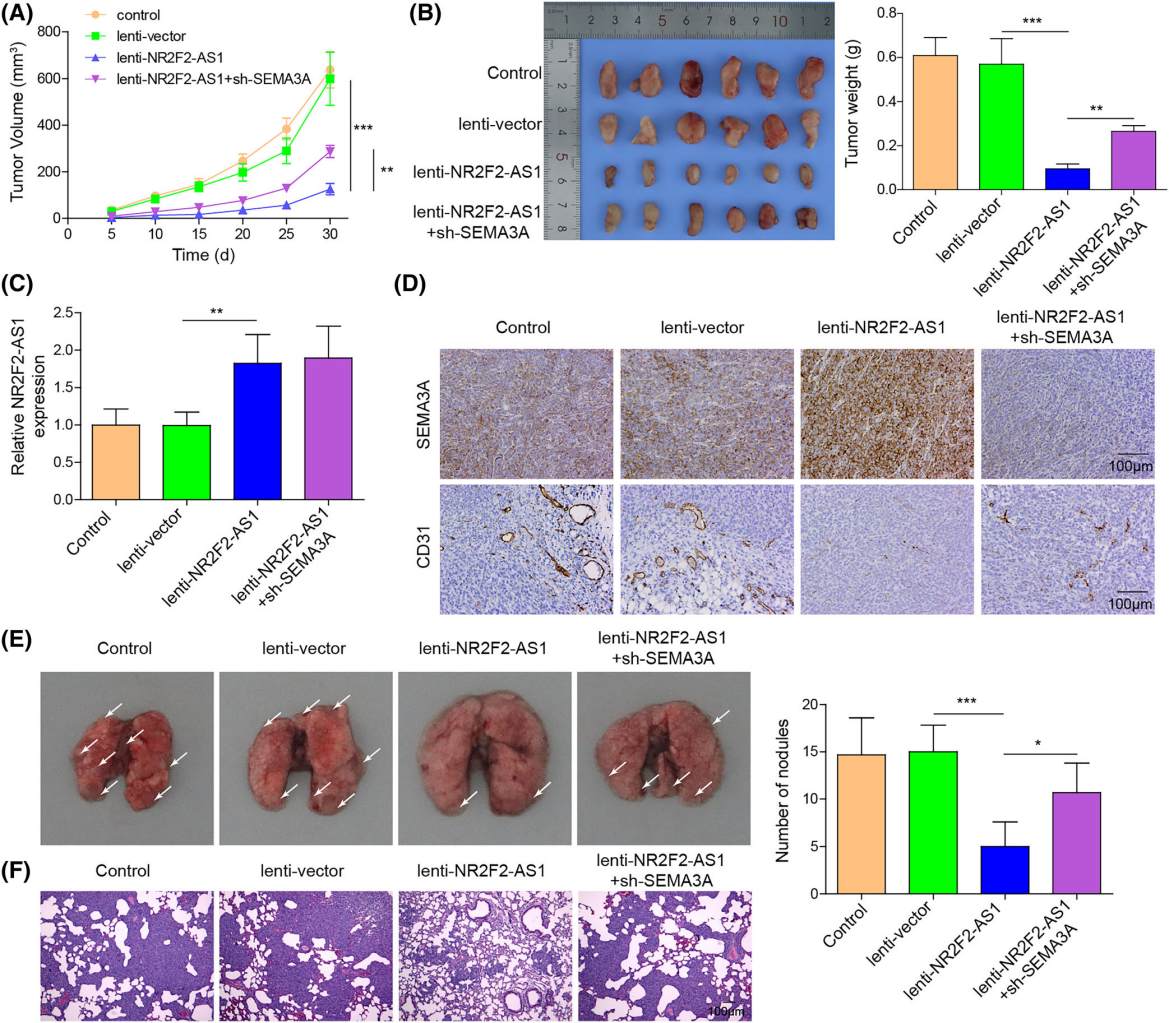
NR2F2‐AS1 impeded tumor growth and metastasis of OSCC in vivo through the miR‐32‐5p/SEMA3A axis. SCC9 cells infected with lentivirus carrying NR2F2‐AS1 or together with sh‐SEMA3A were inoculated into nude mice to induce subcutaneous tumors. After 30 days, tumors were removed. (A and B) Tumor volume and tumor weight were recorded. (C) NR2F2‐AS1 expression was measured using RT‐qPCR. (D) SEMA3A and CD31 expression was evaluated using IHC (scale bar: 100 μm). SCC9 cells infected with lentivirus carrying NR2F2‐AS1 or together with sh‐SEMA3A were injected into nude mice via the tail vein. (E) The images of lungs and statistical bar chart for each group. (F) Pulmonary metastatic nodules were observed by HE staining (scale bar: 100 μm). *p < 0.05, **p < 0.01, ***p < 0.001. HE, hematoxylin and eosin; IHC, immunohistochemistry; OSCC, oral squamous cell carcinoma; RT‐qPCR, quantitative real‐time PCR; SEMA3A, semaphorin 3A.

Subsequently, tumor formation experiments in nude mice were performed to verify the in vitro results. SCC9 cells infected with lentivirus carrying NR2F2‐AS1 or together with sh‐SEMA3A were inoculated into nude mice to induce subcutaneous tumors. As presented in Figure 5A,B, NR2F2‐AS1 overexpression inhibited tumor volume and tumor weight, which was reversed by SEMA3A silencing. Additionally, NR2F2‐AS1 upregulation increased NR2F2‐AS1 and SEMA3A expression and decreased CD31 expression in tumors, whereas SEMA3A knockdown offset NR2F2‐AS1 upregulation‐induced elevation of SEMA3A expression (Figure 5C,D). To probe the influence of the NR2F2‐AS1/SEMA3A axis on pulmonary metastasis, SCC9 cells infected with lentivirus carrying NR2F2‐AS1 or together with sh‐SEMA3A were injected into nude mice via the tail vein. After 30 days, NR2F2‐AS1 overexpression reduced the number of pulmonary metastatic nodules, which was reversed by SEMA3A knockdown (Figure 5E,F). Collectively, these findings indicate that NR2F2‐AS1 upregulation suppressed tumor growth in nude mice by enhancing SEMA3A expression.

## 4 DISCUSSION

Despite advances in tumor therapies, OSCC has a poor prognosis (5‐year survival rate: <50%) because of its high propensity for malignant growth and metastasis.^19^ Tumor characteristics including cell invasion, migration, EMT, and angiogenesis play a key role in OSCC progression.^20^ The present study suggests that NR2F2‐AS1 regulates the miR‐32‐5p/SEMA3A axis to inhibit EMT, migration, and invasion of OSCC cells and the angiogenesis of HUVECs, and to suppress tumor growth and metastasis in mice, thereby alleviating OSCC development.

SEMA3A was the first neuro‐directing factor identified in the SEMA3 family. There is substantial experimental evidence that it acts as a tumor suppressor gene in several tumors.^21^ A previous study documented downregulated expression of SEMA3A in gastric cancer (GC) cells, and SEMA3A overexpression was found to impede GC cell proliferation, migration, invasion, and EMT.^22^ In addition, Yan‐Chun et al. suggested that SEMA3A, negatively mediated by miRNA‐192‐5p, inhibited cell proliferation and metastasis of hepatocellular carcinoma.^23^ A multi‐omics study identified SEMA3A as a prognostic biomarker in squamous cell carcinoma.^24^ SEMA3A, regulated by the Wnt/β‐catenin‐dependent pathway, has been implicated in oral cavity cancer including OSCC.^25^ Furthermore, a prior study indicated that SEMA3A may suppress angiogenesis and tumor growth in mice,^9^ suggesting its potential role in OSCC. In the present study, SEMA3A expression was found to be largely decreased in OSCC cells. Besides, SEMA3A overexpression led to suppression of EMT, migration, and invasion of OSCC cells and angiogenesis of HUVECs, suggesting its potential role in inhibiting malignant attributes of OSCC.

Currently, there are few studies on the upstream mechanism of SEMA3A in tumors, mainly focusing on the effects of miRNA on SEMA3A. Previous studies suggest that SEMA3A is regulated by multiple miRNAs involved in the occurrence and development of tumors. For example, miR‐362 was shown to interact with SEMA3A and silence its expression to promote lung cancer.^8^ miRNA‐192‐5p was found to promote the proliferation and metastasis of hepatocellular carcinoma cells by interacting with SEMA3A to decrease SEMA3A expression.^23^ To further investigate the upstream regulatory molecules of SEMA3A in OSCC, we sought to identify miRNAs that can downregulate targeted gene expression by forming RISC with the targeted gene's mRNA.^26^ There are numerous examples of the mechanism by which the miRNA‐mRNA regulatory axis plays a role in diseases.^27^, ^28^ For instance, miR‐204/CD44 axis, miR‐1247‐3p/FOXO1 axis, and miR‐26a‐5p/SLC7A11 axis have been implicated in NSCLC, bladder cancer, and OSCC, respectively.^28^, ^29^, ^30^ In the present study, using Starbase, miRDB, and miRWalk databases and dual luciferase assay, we identified miR‐32‐5p as the upstream molecule of SEMA3A. Besides, the interaction between miR‐32‐5p and SEMA3A was further validated by dual luciferase assay and RIP assay. Moreover, miR‐32‐5p silencing upregulated SEMA3A expression at mRNA and protein levels. To the best of our knowledge, this is the first study to identify miR‐32‐5p as the upstream regulator of SEMA3A in OSCC.

The lncRNA‐miRNA‐mRNA axis in diseases has been investigated.^14^, ^31^ The lncRNA PWAR6/miR‐577/PHACTR1 axis is involved in regulating the sensitivity of NSCLC to cisplatin.^32^ LncRNA HOXA11‐AS was shown to enhance the malignant attributes of OSCC cells via the miR‐98‐5p/YBX2 axis.^33^ In the present study, NR2F2‐AS1 was screened as the upstream molecule of miR‐32‐5p in OSCC based on literature review, prediction of LncBook and lncRNASNP 2, and RNA pulldown, dual luciferase, and RIP assays. A large body of evidence supports the potential role of lncRNAs as diagnostic biomarkers and therapeutic targets in cancers including OSCC.^34^ For example, lncRNA DUXAP9, lncRNA ENAH‐202, and lncRNA MALAT1 were shown to be related to OSCC progression.^35^, ^36^, ^37^ A previous study reported decreased expression of NR2F2‐AS1 in OSCC and its overexpression impaired the proliferation of OSCC cells, ostensibly via regulating the methylation of miR‐494.^12^ However, there is limited experimental evidence of the role of NR2F2‐AS1 in OSCC. In this study, NR2F2‐AS1 overexpression elevated SEMA3A expression, blocked the EMT process, migration, and invasion of OSCC cells, inhibited angiogenesis of HUVECs, and suppressed tumor growth and metastasis in mice. As anticipated, SEMA3A knockdown abolished NR2F2‐AS1 overexpression‐mediated influence on OSCC cells and mice.

In conclusion, we illustrated that NR2F2‐AS1 modulated the miR‐32‐5p/SEMA3A axis to attenuate EMT, migration, and invasion of OSCC cells, inhibit angiogenesis of HUVECs, and impede tumor growth and metastasis in mice. Our findings may inform further research to identify biomarkers and therapeutic targets for OSCC.

## CONFLICT OF INTEREST STATEMENT

The authors declare no conflicts of interest.

## Supporting information


**Figure S1.** SEMA3A downregulation enhanced the EMT, migration, and invasion of OSCC cells and the angiogenesis of HUVECs. SCC9 and SCC25 cells were transfected with sh‐SEMA3A. (A) SEMA3A, E‐cadherin, N‐cadherin, and Vimentin expression were detected by Western blot assay. (B) Cell migration and invasion were measured using Transwell assay. HUVECs were treated with medium supernatants, which were obtained from OSCC cells with the indicated transfection. (C) Angiogenesis of HUVECs was detected by tube formation assay.

**Table S1.** Target score in miRDB database of different miRNAs.
**Table S2.** Related reports of 17 miRNAs in OSCC.
**Table S3.** 21 lncRNAs with downregulated expression in OSCC.
