# Supplementary figures and images for: LncRNA NR2F2‐AS1 inhibits the progression of oral squamous cell carcinoma by mediating the miR‐32‐5p/SEMA3A axis
Source: Kaohsiung J Med Sci. 2024 Aug 23;40(10):877–89. doi: 10.1002/kjm2.12888 (PMC11895586; doi:10.1002/kjm2.12888)

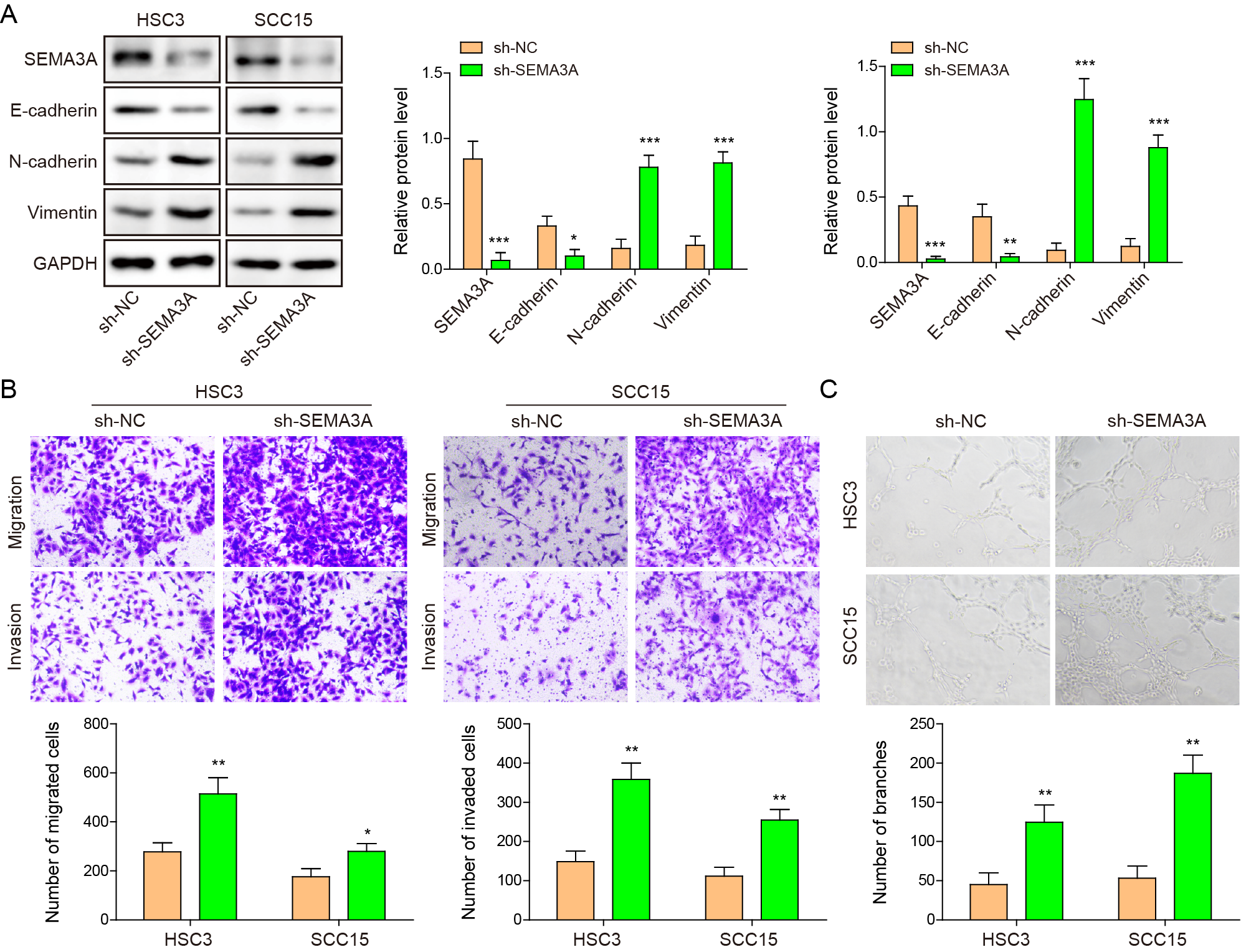

Supplement: Supplementary file 1 — Figure S1. SEMA3A downregulation enhanced the EMT, migration, and invasion of OSCC cells and the angiogenesis of HUVECs. SCC9 and SCC25 cells were transfected with sh‐SEMA3A. (A) SEMA3A, E‐cadherin, N‐cadherin, and Vimentin expression were detected by Western blot assay. (B) Cell migration and invasion were measured using Transwell assay. HUVECs were treated with medium supernatants, which were obtained from OSCC cells with the indicated transfection. (C) Angiogenesis of HUVECs was detected by tube formation assay. [file KJM2-40-877-s001.tif]
